# Considerations in Flap Selection for Soft Tissue Coverage of the Hand and Digits

**DOI:** 10.3390/jcm15010013

**Published:** 2025-12-19

**Authors:** Piotr Węgrzyn, Marta Jagosz, Maja Smorąg, Szymon Manasterski, Michał Chęciński, Paweł Stajniak, Jędrzej Króliński, Patryk Ostrowski, Paweł Poznański, Dorota Kamińska, Ahmed Elsaftawy

**Affiliations:** 1 Department of Plastic and Hand Surgery, St. Hedwig of Silesia Hospital, 55-100 Trzebnica, Poland; 2 Department of Anatomy, Jagiellonian University Medical College, 31-008 Krakow, Poland; 3 Youthoria, Youth Research Organization, 30-363 Krakow, Poland; 4 Faculty of Medicine, Wroclaw University of Science and Technology, 50-370 Wroclaw, Poland

**Keywords:** perforator flaps, thenar flap, reconstruction, upper limb

## Abstract

**Background/Objectives**: The goal of reconstructive hand surgery is to achieve both functional and aesthetic restoration. The primary aim of this study is to perform a detailed, practice-oriented evaluation of perforator-based and local flaps for soft-tissue reconstruction of the hand and digits, focusing specifically on their functional reliability, anatomical consistency, complication profile, and aesthetic integration in a real-world, high-complexity referral population. **Methods**: This retrospective single-center study included 37 patients with soft tissue defects of the hand that required flap coverage between September 2021 and September 2024. The study assessed patient demographics, defect characteristics, flap selection, surgical techniques, and outcomes including satisfactory soft tissue coverage, functional results and occurrence of complications. Various perforator flaps were analyzed, including the dorsal metacarpal artery flap, reverse radial forearm flap, reverse posterior interosseous artery flap, reverse homodigital and heterodigital island flaps, and the thenar flap. **Results**: Satisfactory soft tissue coverage was achieved in 35 out of 37 patients. One case involved partial distal flap necrosis, and another presented with Foucher flap failure. The remaining flaps demonstrated stable integration, preserved perfusion, and durable soft-tissue coverage with satisfactory contour and pliability. Functional outcomes were favorable, with restoration of joint mobility and absence of secondary deformities. **Conclusions**: This study supports the continued use of perforator and local flaps in upper extremity reconstruction, emphasizing the need for individualized planning to optimize the outcomes.

## 1. Introduction

The primary objective of reconstructive hand surgery is the restoration of function while maintaining an aesthetically acceptable appearance. The hand is a highly visible and functionally complex organ, making both aspects equally important [1]. Despite significant advancements in microsurgical techniques, an optimal strategy for soft tissue coverage remains a subject of discussion. The literature has extensively documented various flap techniques; however, there is still an ongoing debate regarding the ideal balance between function, aesthetic outcomes, and donor-site morbidity [2,3,4].

A systematic approach to hand defect coverage begins with thorough wound debridement, performed under tourniquet control to ensure the removal of all nonviable tissues while preserving vital structures. Once debridement is complete, the reconstructive ladder concept helps guide the decision-making process, categorizing defects into those that are simple, suitable for direct closure, skin grafting, the use of a dermal substitute, or secondary wound healing; and complex, requiring flap-based reconstruction [5]. For cases necessitating soft tissue coverage beyond a skin graft, perforator-based flaps and local pedicled flaps have gained prominence as a reliable solution due to their ability to provide pliable, well-vascularized coverage with minimal donor-site morbidity [6]. Alternatives to these options include free flaps and distant pedicled flaps.

In this study, we present our experience using perforator flaps for reconstruction of palm and dorsum of the hand, as well as thumb and digit defects, emphasizing their efficacy in achieving functional recovery and aesthetic integration. We analyze the indications, technical aspects, complications, and outcomes associated with the following flaps:

- Dorsal Metacarpal Artery Flaps;
- Reverse Radial Forearm Flap;
- Reverse Posterior Interosseous Artery (PIA) Flap;
- Reverse Homodigital and Heterodigital Island Flaps;
- Thenar Flap.

## 2. Materials and Methods

### 2.1. The Patient Selection and Study Design

This retrospective study included 37 patients who underwent soft tissue reconstruction for hand defects at Department of Plastic and Hand Surgery, St. Hedwig of Silesia Hospital, Trzebnica, Poland, between September 2021 and September 2024. All patients required soft tissue coverage beyond direct closure or skin grafting. Inclusion criteria consisted of:

- Patients with soft tissue defects of the hand requiring flap coverage;
- Availability of adequate recipient area and donor site possibility;
- No contraindications to regional flap surgery;
- At least 6-month follow-up.

A total of 37 patients (32 males, 5 females) with a mean age of 47 years (range: 26–76 years) were included. The majority (94.6%) of defects resulted from traumatic injuries (31 sharp/crush injuries, 3 burns, 1 dog bite), while 2 cases were non-traumatic (Dupuytren’s contracture and diabetic thumb infection).

The majority of our patients were referred from external centers after unsuccessful primary management (*n* = 25; 67.6%), often presenting with infected or devitalized wounds, necrosis following replantation, chronic scarring or contractures, and traumatic wound beds resulting from previous interventions, as well as burns (thermal, electrical, or chemical).

### 2.2. Wound Debridement

Surgical debridement of the defect site was performed as soon as possible—ideally within 24 h since the emergence of a defect—necrotic tissues, contaminated substances adhering to the wound site, and foreign bodies were removed from the surgical wound. If there were concerns about spread of infection or the appearance of additional areas of tissue necrosis, coverage of the defect was postponed for 1–2 days to ensure the good condition of the local tissues, while the defect was left open and covered with an occlusive dressing In cases of chronic defects, our goal has been to achieve one-stage surgery defect management that included both debridement of the defect and definitive closure using a flap procedure. If there were any concerns regarding the feasibility of definitive management of the defect, the coverage is postponed for 1–2 days, similar to the approach used for acute post-traumatic defects. In cases where clinical indications suggested infection, microbiological and laboratory examinations were conducted to identify the pathogen and guide targeted antibiotic therapy in addition to surgical treatment [7,8].

### 2.3. Preoperative Planning and Flap Selection

Preoperative assessment included hand vascular mapping with Doppler ultrasonography to evaluate the presence and course of critical perforators. Flap selection was based on defect size, location, and tissue requirements. Flaps were harvested according to standard anatomical landmarks and microvascular techniques. The reconstructive approach followed the reconstructive elevator principle, prioritizing local flaps before considering regional or distant options [9]. Our target was to perform definite flap coverage within 72 h from debridement [10,11].

### 2.4. Surgical Techniques

Each flap type was performed according to well-established techniques:

#### 2.4.1. Reverse Posterior Interosseous Artery (PIA) Flap

**Figure 1:**
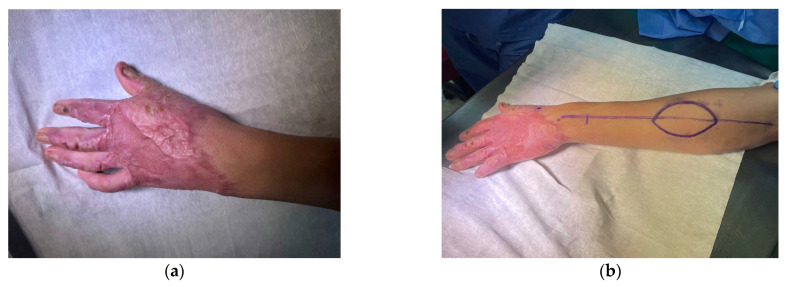

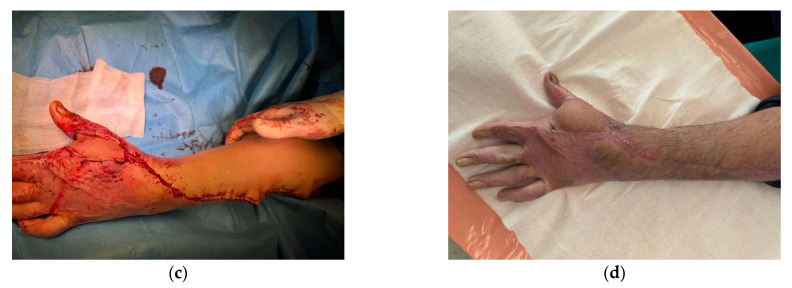
(**a**) A 26-year-old male with contracting scarring of the first webspace due to thermal burn. (**b**) PIA flap design; (**c**) Early post-operative effect; (**d**) Result 3 months after surgery.

The reverse posterior interosseous artery (PIA) flap is supplied by perforators arising from the PIA, which are situated in the intermuscular septum between the fifth and sixth extensor tendon compartments—specifically between the extensor carpi ulnaris and extensor digiti minimi—in the middle third of the forearm. The pivot point is situated roughly 3 cm proximal to the distal radioulnar joint, at the site where the PIA forms an anastomosis with the anterior interosseous artery. This flap can cover defects on the dorsum of the hand such as the MCP joints defects or the first webspace [12,13,14]. In our study this flap was used to cover 2 medium-size dorsal hand defects and in one case of contracting scar tissue of first webspace (Figure 1).

#### 2.4.2. Dorsal Metacarpal Artery (DMA) Flap

The Kite, or Foucher, flap is a commonly employed technique for reconstructing and preserving thumb integrity after injury. It is derived from the first dorsal metacarpal artery flap. The name “Foucher flap” honors the French surgeon Guy Foucher, who first performed and reported this method in 1979 [15]. The first dorsal metacarpal artery flap arises from the radial artery and courses alongside the second metacarpal of the index finger, lying superficial to the fascia of the first dorsal interosseous muscle.

Modifications of this flap may include the dorsal sensory branch of the radial nerve, the second metacarpal, the extensor indicis proprius muscle, and the first dorsal interosseous muscle for management of more complex defects. Most frequently this technique is used for management of both dorsal and palmar thumb defects as well as defects of the first webspace [16].

Variations in this flap, including the reverse or extended metacarpal artery flaps, permit successful coverage distal to the interphalangeal joint via the palmar-dorsal anastomosis located at the level of the proximal phalanx rather than the metacarpal neck [17,18].

**Figure 2:**
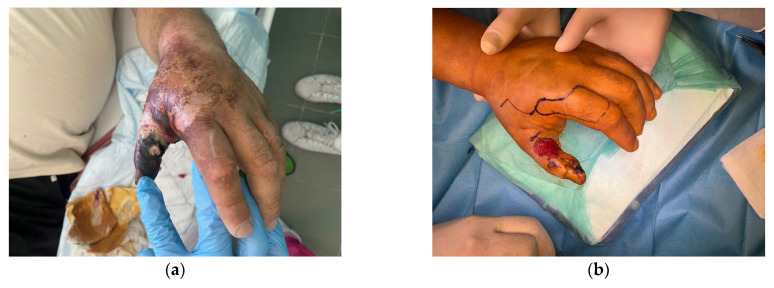

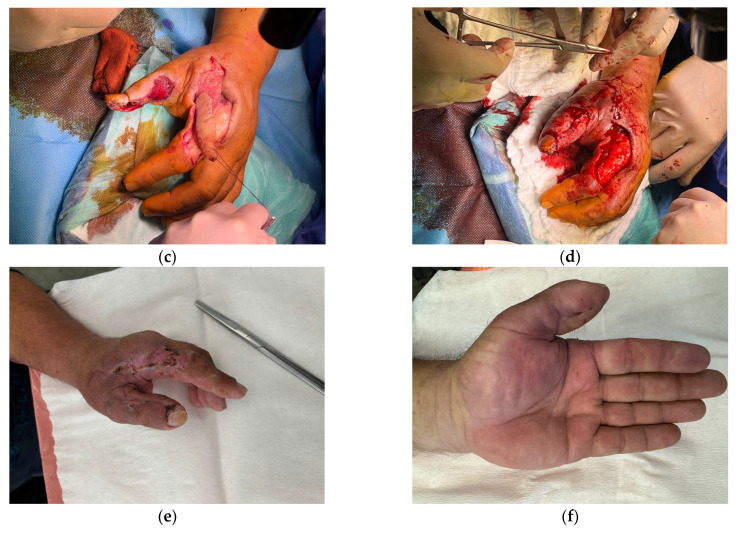
(**a**) A 56-year-old man presented with thumb infection with soft tissue necrosis in course of untreated diabetes; (**b**) Foucher flap design; (**c**) Flap raising; (**d**) Insetting of the flap (**e**,**f**) Results 6 weeks post-surgery.

This flap was used in 9 patients with thumb soft tissue defects (Figure 2).

**Figure 3:**
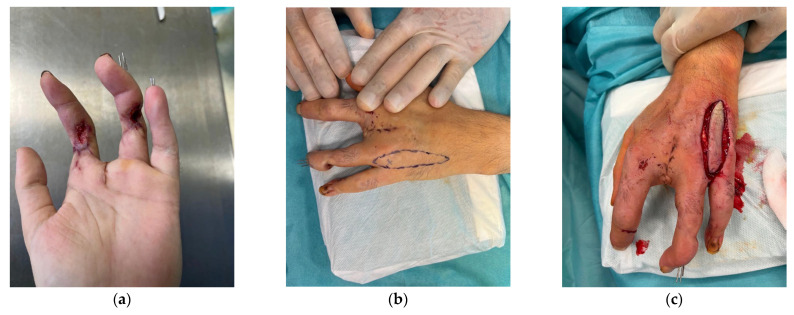

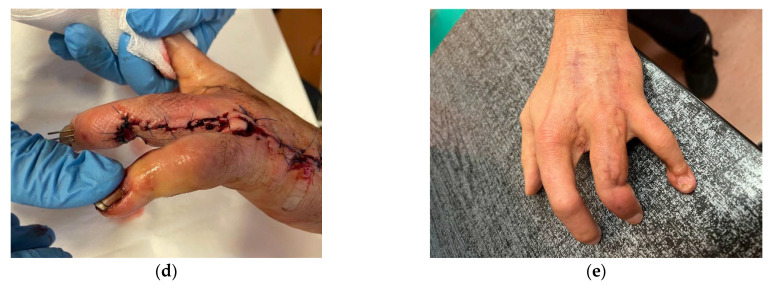
(**a**) A 44-year-old male presented with PIP 2 and PIP 4 defects due to industrial injury resulting in third digit amputation; (**b**) DMCA flap design; (**c**) Flap raising; (**d**) Early post-operative outcome (**e**) Post-operative outcome 12 weeks after surgery.

Use of the second dorsal metacarpal artery, a branch of the dorsal carpal arch, enables coverage of ulnar defects on the dorsal aspect of the hand and fingers. Those flaps can be found in the literature under the name Maruyama or Quaba flap. Microsurgical expertise is necessary to raise these flaps, and from a technical standpoint the operation is of moderate difficulty [16,19,20,21]. This flap was used in 4 patients with PIP region soft tissue defects of the digits (Figure 3).

#### 2.4.3. Reverse Radial Forearm (RRF) Flap

Flaps based on the radial artery are highly versatile and can be used to reconstruct a wide variety of hand defects. The reversed radial fasciocutaneous or adipofascial flap is most commonly employed for coverage of defects on the dorsal or palmar aspects of the hand. In addition, several modifications have been reported in the literature, including its use as an osteofasciocutaneous flap, a cutaneous perforator-based flap, and as a free flap [18,22]. The palmaris longus tendon can be included in the flap design along with the lateral antebrachial cutaneous nerve to provide both motor function and sensory innervation. The traditional RRF flap is dependent on reverse flow from the ulnar artery traveling through the deep palmar arch and ultimately supplying the radial artery. Consequently, an abnormal Allen test prior to surgery serves as an absolute contraindication to utilizing this technique. For post-traumatic reconstruction of composite thumb defects, it may be used as radial forearm flap covering the skeletonized thumb fragment or other osseous transplant in the place of a thumb [23].

**Figure 4:**
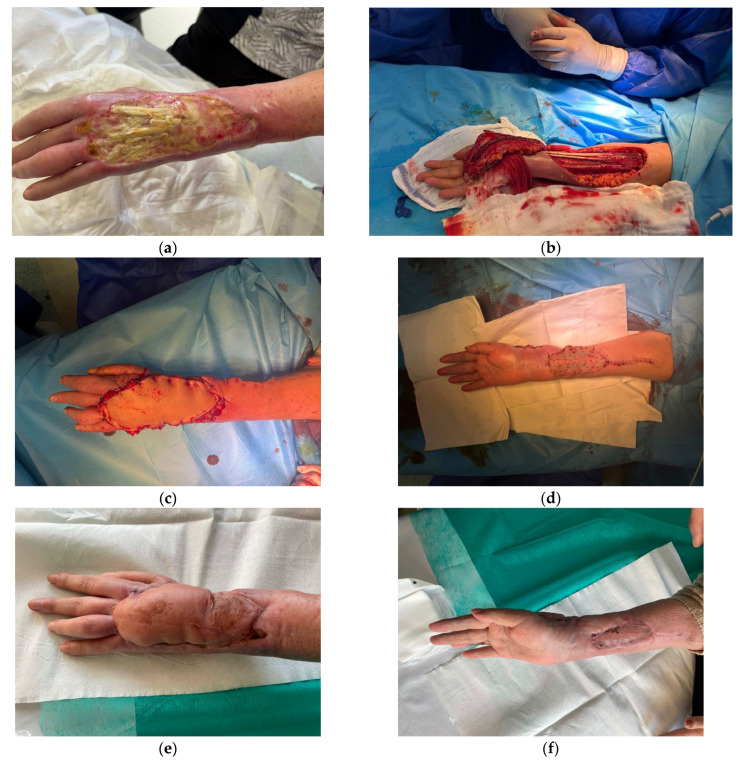
(**a**) A 76-year-old female suffered chemical burn which resulted in dorsal hand defect exposing extension tendons; (**b**) Reverse radial artery flap raising; (**c**) Immediate post-operative result; (**d**) Donor site covered with split-thickness skin graft; (**e**) Post-operative outcome 8 weeks after surgery; (**f**) Donor site 8 weeks after surgery.

The flap is oriented along the course of the radial artery, with the skin paddle preferably positioned over the middle third of the forearm. A common pitfall is positioning the skin paddle too proximally, as most perforators are located in the distal third of the forearm. The flap can effectively cover defects measuring up to 10–20 cm, extending to the proximal interphalangeal joint. Although the dissection of the RRF flap is relatively straightforward and anatomical variation is minimal, some surgeons express reluctance to use this flap due to the requirement for sacrificing the radial artery. If used as a proximally based free flap, careful closure and imbrication of muscle over the distal flexor carpi radialis tendon is imperative to reduce donor morbidity and improve skin graft take to the donor site [24,25]. In the material, this flap was used to cover 5 medium-to large dorsal hand defects as well as 2 palmar hand defects and 2 thumb defects (Figure 4).

#### 2.4.4. Reverse Homodigital and Heterodigital Island Flap

**Figure 5:**
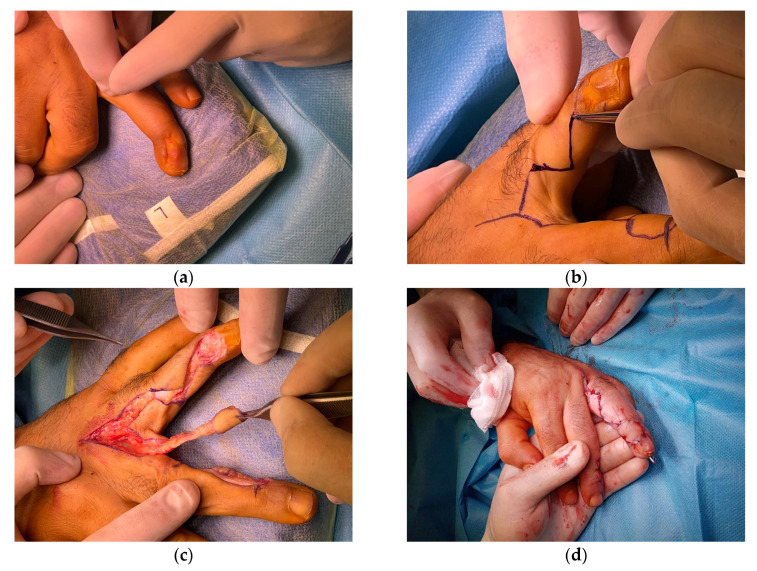
(**a**) A 37-year-old male with dorsal IV finger DIP joint defect with a fistula due to open fracture; (**b**) Flap design; (**c**) Island finger reverse artery flap raising; (**d**) Early post-operative outcome.

This is a versatile option for coverage of both dorsal and palmar defects of the fingers, with the maximum size of 2.5 cm to 3 cm. The main advantage of homodigital island flap technique is that all defects are confined to one finger, preserving other fingers uninjured. An axial pattern flap derives its blood supply from reverse flow of the radial or ulnar digital artery. Three anastomotic sites link the radial and ulnar digital arteries: at the proximal cruciate ligament, at the distal cruciate ligament, and at the fingertip just distal to the profundus tendon insertion. Any one of these connections can provide sufficient perfusion for the flap. This flap is technically demanding, requiring meticulous dissection of the nerve from the artery. Due to the necessity of retracting the nerve, most patients may experience a transient neurapraxia postoperatively [26]. The flap can also be planned as a heterodigital flap to reconstruct defects of adjacent fingers. Similarly to the reverse-flow homodigital flap, the heterodigital flap offers considerable freedom in its arc of rotation, making it suitable for coverage of both volar and dorsal finger defects [27] (Figure 5).

#### 2.4.5. Thenar Flap

**Figure 6:**
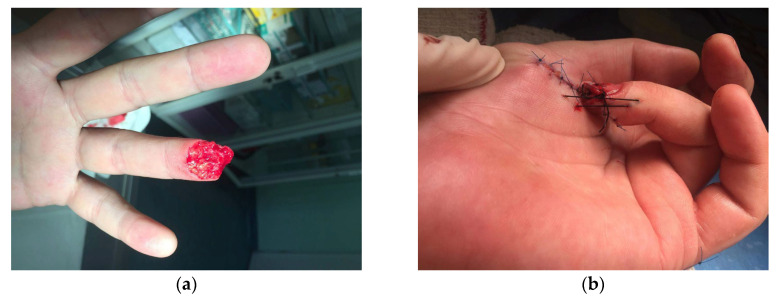

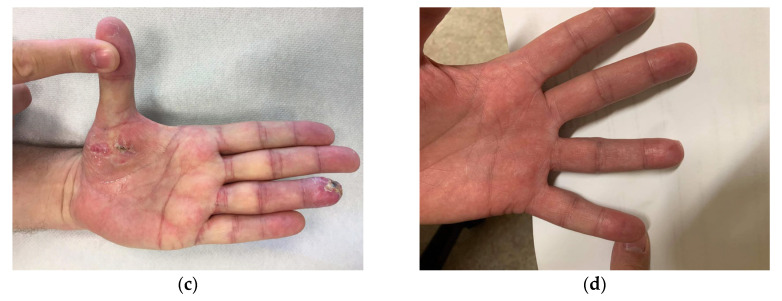
(**a**) A 53-year-old male sustained fingertip amputation of the fourth digit due to mechanical injury; (**b**) Early post-operative outcome of covering the defect with thenar flap; (**c**) Outcome 6 weeks after surgery; (**d**) Outcome 3 months after surgery.

Gatewood originally introduced the thenar flap technique in 1926, and since that time it has appeared in the literature with various modifications for the reconstruction of fingertip injuries and amputations [28] (Figure 6).

Table 1 shows the individual flaps that were used in our department to cover soft tissue defects of the hand region between September 2021 and September 2024.

### 2.5. Postoperative Management

Post-operative treatment consisted of regular flap monitoring (at least once every 6 h) for 5 days, subcutaneous enoxaparin administration twice daily in therapeutic dosage and non-adhesive dressing changes. In cases of simpler reconstruction techniques, e.g., thenar flap, patients were discharged from the hospital on the first day after surgery, and follow-up appointments were conducted on the third and seventh days post-surgery. Flap division was performed on 14th day POD Sutures were removed on the 14th post-operative day and early physical therapy was started as soon as viability of the flap was secured [29,30]. Table 2 summarizes the postoperative management of the operated patients.

## 3. Results

A total of 37 patients underwent soft-tissue reconstruction of the hand and digits using a variety of regional and local flaps. The cohort represented a spectrum of post-traumatic and post-necrotic tissue losses involving both the dorsal and volar aspects of the hand, thumb, and fingers.

The etiologies of the defects were predominantly mechanical trauma (*n* = 25; 67,5%), followed by secondary necrosis following crushing injury or failed replantation (*n* = 7; 18,9%), burns of thermal, electrical, or chemical origin (*n* = 3; 8,1%), infection associated with diabetes mellitus (*n* = 1; 2,7%), and recurrent Dupuytren’s contracture (*n* = 1; 2,7%).

The anatomic distribution of defects included dorsal and palmar defects of the hand (*n* = 9), thumb (*n* = 11), fingertips (*n* = 9), and phalangeal or web space defects (*n* = 8). Flap selection was individualized according to defect location, size, and depth, as well as donor-site characteristics and vascular anatomy.

The major complication rate (requiring secondary surgical procedure) was 5.4%. One patient developed partial distal flap necrosis following a dog bite, managed successfully with surgical debridement and split-thickness skin graft. Another case, a diabetic patient with an infected volar thumb defect reconstructed using a Foucher flap, experienced total flap failure necessitating secondary coverage. The minor complications consisted of transient neuropraxia in patients treated with island digital flaps that resolved within 3 months without any intervention. All of the complications mentioned were classified as early complications, occurring within four weeks following the procedure.

In all remaining patients, the flaps demonstrated stable integration, preserved perfusion, and durable soft-tissue coverage with satisfactory contour and pliability. Functional outcomes were favorable, with restoration of joint mobility and absence of secondary deformities. Functionally, most patients were able to resume hand mobility by postoperative day 14, following our standardized early-mobilization protocol. Aesthetic integration was satisfactory across flap types. These results suggest that perforator-based reconstruction remains robust even in suboptimal wound environments, supporting its use in tertiary referral settings where reconstructive demands exceed what is typically described in the literature.

### 3.1. Flap Selection and Outcomes

Flap selection and outcomes are presented in Table 3.

### 3.2. Complications

Postoperative complications are listed below:

- 1 case of partial flap necrosis (managed with debridement and split-thickness skin graft—STSG);
- 1 case of total flap failure (necessitating secondary reconstruction);
- Transient neuropraxia in reverse digital artery flaps (resolved within 3 months).

## 4. Discussion

The reconstructive elevator concept suggests that the simplest option for coverage might not always be the best in terms of form and function, emphasizing the need for individualized reconstructive planning [31].

The selection of appropriate flap techniques for soft tissue defects of the hand requires a nuanced understanding of various factors, including defect characteristics, patient condition, and specific functional and aesthetic requirements. Early reconstruction of soft tissue defects in the hand has been associated with favorable outcomes, facilitating the patient’s return to high functional levels. Our findings reinforce the effectiveness of perforator flaps in achieving stable soft tissue coverage while preserving hand function. Early reconstruction was associated with lower infection rates and improved functional recovery, aligning with literature emphasizing early intervention (within 72 h) for optimal outcomes [10,11]. It is crucial for the hand surgeon to recognize that multiple reconstructive options often exist for a given defect. A thorough assessment of tissue type, as well as functional and aesthetic goals, is essential to achieve optimal outcomes. The “like with like” principle, which advocates for replacing lost tissue with similar tissue in appearance and function, is fundamental in hand reconstruction [2,3,4,5]. Additionally, flap thinning is often necessary to ensure that the reconstructed tissue closely matches the native hand tissue in thickness and pliability, thereby enhancing both functional and cosmetic results [3,5,13]. The best functional and aesthetic results in these patients are achieved with free flaps coverage; however, when early free flap transfer is not feasible due to patient instability or other contraindications, alternative options such as local and regional flaps should be considered. Local flaps, which consist of skin and subcutaneous tissue harvested from a site adjacent to the defect while maintaining their intrinsic blood supply, can be reliable sources for soft tissue replacement in the hand [4,6]. We emphasize achieving favorable reconstructive outcomes in compromised tissue beds, scenarios where perforator-based reconstructions are frequently avoided in the literature. Highlighting the reliability of these flaps in such adverse conditions provides clinically significant, practice-modifying information. A critical comparison of our results with existing literature provides insights into the relative advantages and limitations of each technique.

### 4.1. Reverse Posterior Interosseous Artery (PIA) Flap

The reverse PIA flap is particularly advantageous for dorsal hand defects due to its anatomical proximity and reliable blood supply. Our positive outcomes with this flap are consistent with those reported in the literature. Nonetheless, the PIA flap’s utility may be limited by anatomical variations and a steep learning curve, necessitating meticulous preoperative planning and surgical expertise [12,13,14].

### 4.2. Dorsal Metacarpal Artery Flaps

The dorsal metacarpal artery (DMA) flap is a versatile option for resurfacing soft-tissue defects of the fingers. Our findings align with those of Sebastin et al., who demonstrated that the DMA perforator flap is effective for defects proximal to the fingertip, offering reliable coverage with satisfactory aesthetic and functional outcomes. However, donor site morbidity, such as the need for skin grafting (if harvested flap is more than 3.5 cm in width), has been reported. Innovative techniques, like the use of digital dorsal advancement flaps to repair donor sites, have been proposed to address these challenges [16,17,19,20,21].

### 4.3. Reverse Radial Forearm Flap

The reverse radial forearm flap has been a cornerstone in reconstructive surgery due to its robust vascularity and pliability. Our results corroborate existing literature, indicating that this flap provides durable coverage for extensive soft tissue defects. However, donor site morbidity remains a concern. Alternative approaches, such as the radial artery perforator flap, have been explored to mitigate these issues by preserving the radial artery, thereby reducing complications; however, it has greater limitations resulting from the length of the pedicle [24,25].

### 4.4. Reverse Homodigital and Heterodigital Island Flaps

Reverse digital artery island flaps are widely employed for fingertip reconstructions, offering good aesthetic and functional results. Our experience aligns with studies demonstrating that these flaps provide reliable coverage with satisfactory sensory recovery [26]. However, donor site morbidity, such as the need for skin grafting if donor side defect is too big to be covered primarily, has been reported. Innovative techniques, like the use of digital dorsal advancement flaps to repair donor sites, have been proposed to address these challenges [27].

### 4.5. Thenar Flap

The thenar flap is a traditional method for reconstructing fingertip and pulp defects, particularly in young patients. Our results with the thenar flap are comparable to those in existing literature, showing favorable outcomes in terms of flap survival and functional recovery. However, limitations such as joint stiffness and the need for a two-stage procedure should be considered when selecting this technique [28].

## 5. Limitations

This study has several limitations. Its retrospective design may constrain the ability to draw definitive conclusions regarding causality and treatment efficacy. With only 37 patients included, the sample size may be insufficient for generalizing findings to broader populations. Additionally, as a single-center study, the applicability of the results may be limited to specific settings. Variability in patient demographics, defect types, and surgical techniques complicates objective comparisons between functional and aesthetic outcomes. Future studies would benefit from the use of objective measures or standardized scoring systems to enhance evaluation.

## 6. Conclusions

Perforator flaps remain a cornerstone in reconstructive hand surgery, offering functional and aesthetic advantages. While no single flap is universally superior, an individualized approach based on defect characteristics, patient factors, and surgeon expertise optimizes outcomes. Future research should focus on long-term functional recovery and comparative studies between perforator flaps and alternative reconstructive options.

## Figures and Tables

**Table 1:** Type of flaps used to cover soft tissue defects in the hand area at the Department of Plastic and Hand Surgery, St. Hedwig of Silesia Hospital, Trzebnica, Poland, (2021–2024).

Type of Flap	Indication	No. of Patients
Reverse posterior interossous artery (PIA) flap	Dorsal hand defects and first web space contractures	3
Dorsal Metacarpal Artery (DMA) flap	Thumb and digit defects	9
Reverse radial forearm (RRF) flap	Large dorsal and palmar hand and thumb defects	3
Reverse homodigital and heterodigital island flaps	Digital defects	3
Thenar flap	Fingertip reconstructions	9

**Table 2:** Postoperative Management of the operated patients.

Type of Management	Period
Systemic anticoagulation with 5000 IU heparin intravenously	Intraoperatively
Enoxaparin (1 mg/kg) subcutaneously	Twice daily for 5 days
Regular flap monitoring every 6 h	Every 6 h for 5 days
Non-adhesive dressing changes	Everyday
Suture removal	14th POD
Early mobilization	From 14th POD

POD—post-operative day.

**Table 3:** Flap selection and outcomes.

Age	Sex	Tissue Defect	Type of Flap	Cause of Defect	Complications
36	F	dorsal hand defect	PIAF	necrosis following crushing injury	none
43	M	MCP II defect	PIAF	mechanical injury	none
26	M	first webspace contracting scarring	PIAF	thermal burn	none
36	M	PIP II defect	DMCAF	mechanical injury	none
26	M	PIP II defect	DMCAF	mechanical injury	none
32	M	PIP III defect	DMCAF	mechanical injury	none
44	M	PIP IV defect	DMCAF	mechanical injury	none
76	F	dorsal hand defect	RAF	dog bite	partial flap necrosis
39	M	dorsal hand defect	RAF	mechanical injury	none
32	M	volar thumb defect	RAF	necrosis following replantation	none
43	M	thumb distal phalanx degloving	RAF	mechanical injury	none
57	M	dorsal hand defect	RAF	crushing injury	none
52	M	contracting scar tissue	RAF	recurrent Dupuytren’s disease	none
34	M	complex soft tissue defect of the hand	RAF	high-voltage electric burn	none
76	F	dorsal hand defect	RAF	chemical burn	none
44	M	dorsal hand defect	RAF	necrosis after mechanical injury	none
42	M	thumb IP defect	Foucher	mechanical injury	none
67	M	volar thumb defect	Foucher	necrosis following replantation	none
56	M	volar thumb defect	Foucher	infection due to diabetes	none
67	M	volar thumb defect	Foucher	infection after mechanical injury	flap failure
42	M	volar thumb defect	Foucher	necrosis following subtotal amputation	none
46	M	thumb IP defect	Foucher	mechanical injury	none
52	M	volar thumb defect	Foucher	mechanical injury	none
43	F	dorsal thumb defect	Foucher	necrosis following injury	none
63	M	thumb IP defect	Foucher	mechanical injury	none
63	M	DIP III defect	finger	mechanical injury	none
37	M	DIP IV defect with fistula	finger	mechanical injury	none
49	M	DIP II and DIP III defects	finger	mechanical injury	none
32	M	II digit fingertip defect	thenar flap	mechanical injury	none
43	M	II digit fingertip defect	thenar flap	mechanical injury	none
37	M	III digit fingertip defect	thenar flap	mechanical injury	none
45	M	II digit fingertip defect	thenar flap	mechanical injury	none
47	M	III digit fingertip defect	thenar flap	mechanical injury	none
58	M	III digit fingertip defect	thenar flap	mechanical injury	none
53	M	IV digit fingertip defect	thenar flap	mechanical injury	none
48	M	II digit fingertip defect	thenar flap	mechanical injury	none
53	F	II digit fingertip defect	thenar flap	mechanical injury	none

## Data Availability

The original contributions presented in this study are included in the article. Further inquiries can be directed to the corresponding author.

## Abbreviations

The following abbreviations are used in this manuscript:

PIA	posterior interosseous artery
PIAF	posterior interosseous artery flap
DMA	dorsal metacarpal artery
DMCAF	dorsal metacarpal artery flap
RRF	reverse radial flap
POD	post-operative day
RAF	radial artery flap
MCP	metacarpophalangeal (joint)
PIP	proximal interphalangeal (joint)
DIP	distal interphalangeal (joint)
STSG	split-thickness skin graft
